# IL-2 Receptor Expression in Renal Cell Carcinoma Cells: IL-2 Influences Cell Survival and Induces Cell Death

**DOI:** 10.3390/cimb47100830

**Published:** 2025-10-09

**Authors:** Sophie Grigolo, Isabelle Fellay, Luis Filgueira

**Affiliations:** Anatomy, Section of Medicine, University of Fribourg, 1700 Fribourg, Switzerland; sophie.grigolo@unifr.ch (S.G.); isabelle.fellay@unifr.ch (I.F.)

**Keywords:** renal cell carcinoma, interleukin-2, IL-2Rα, IL-2Rβ, IL-2Rγ subunits

## Abstract

Renal cell carcinoma (RCC) is the most common form of kidney cancer in adults. Immunotherapy, such as the application of interleukin-2 (IL-2), is a crucial treatment. It is known that IL-2 is involved in the upregulation of the anti-tumor immune response; however, a direct action of IL-2 on RCC cells has not yet been demonstrated. In this project, we aimed to investigate the expression and the functionality of the IL-2Rα, IL-2Rβ, and IL-2Rγ subunits on the four human RCC cell lines A-498, ACHN, Caki-1, and Caki-2. The expression of the three subunit genes was investigated via PCR, agarose gel of PCR products, Western blot, and flow cytometry. IL-2R functionality was assessed in RCC cells cultured with or without rhIL-2 using MTT and BrdU assays to investigate cell viability and proliferation; LDH assays, Live-or-Dye staining, and Annexin V/PI staining to study cell death; and Western blot to detect apoptotic markers, cleaved PARP, and cleaved caspases 3 and 9. Expression of IL-2Rα, IL-2Rβ, and IL-2Rγ subunits in the four cell lines was observed at the protein level with Western blot. Flow cytometry confirmed the cell-surface expression of IL-2Rα, IL-2Rβ, and IL-2Rγ subunits. In addition, we observed that rhIL-2 influenced cell survival/proliferation and cell death, depending on the cell line. We conclude that IL-2R is functional in RCC cells and that rhIL-2 could be used as a therapeutic option to act directly on RCC cells. However, further studies are required to elucidate the signaling pathways triggered by the IL-2-receptor binding on RCC cells.

## 1. Introduction

Kidney cancer is the 14th most common cancer worldwide [1]. It is the sixth most prevalent cause of cancer-related death in men and the tenth in women, accounting for 5% and 3% of malignancies, respectively. Following prostate and bladder cancer, kidney cancer is the most frequent and lethal malignant urological disease [2]. More than 90% of kidney cancers are classified as renal cell carcinoma (RCC) type [3,4].

The most prevalent RCC subtypes are clear cell RCC (ccRCC) and papillary RCC (pRCC), accounting for about 75% and 10% of cases, respectively [5].

In the early stages, RCC is generally a silent, asymptomatic disease [6]. More than 50% of renal masses are diagnosed incidentally during thoracoabdominal imaging performed for unrelated problems. The onset of the symptom triad (hematuria, flank pain, and abdominal mass) is typically associated with an advanced or metastatic form of RCC [7,8].

Nephrectomy, partial or radical, represents the standard therapy addressed to locally advanced RCC patients. Cytoreductive surgery and metastasectomy can also be performed in the metastatic forms of RCC [9].

In addition to surgery, most RCC patients are candidates for systemic therapies, such as immunotherapy (immune checkpoint inhibitors and cytokines) and target therapy (tyrosine kinase inhibitors), including patients with advanced disease and late diagnosis, high-risk recurrence patients, and patients unfit for surgery due to comorbidities or kidney failure [10,11,12].

In 1992, the U.S. Food and Drug Administration approved high-dose IL-2 (HD IL-2) as the first immunotherapy for treating various cancers, including renal cancer [13].

The systemic administration of HD IL-2 therapy (Aldesleukin) stimulates the activation of the immune cells, especially the effector T (T_eff_) cells and NK cells, which are anti-tumor cytotoxic cells. These IL-2-stimulated immune cells, also defined as lymphokine-activated killer cells, secrete TNFα, IFNγ proteins, and the components of the cytolytic granules, including perforin, granzyme A, and granzyme B, which may kill RCC cells. Perforins form pores in the membrane of the cancer cells. These pores cause osmotic lysis and damage the mitochondria. On the other hand, the pores represent a passageway for granzymes, which trigger programmed cell death pathways once inside the cancer cells [3].

Since IL-2 has a short half-life due to its low molecular weight of 15.5 kDa, it must be administered at high doses to be effective. Unfortunately, HD IL-2 induces severe side effects in patients with RCC. Occurrences of capillary leak syndrome and reduced neutrophil function, along with fever, hypotension, and vomiting, are often observed [14,15,16].

For these reasons, the administration of HD IL-2 therapy is restricted to patients with adequate organ function and whose physiological functions are closely monitored during the treatment administration [17].

A further significant challenge linked to IL-2 immunotherapy involves the stimulation of the regulatory T (T_reg_) cells, which promote suppression of the anti-tumor immune responses. The expression of the trimeric IL-2 receptor complex (IL-2Rαβγ) underlies the high-affinity binding of IL-2 to these tumor-protecting immune cells [15].

Over the years, IL-2 has been administered in combination with other immunotherapies, such as pembrolizumab (anti-programmed cell death protein 1 antibody) or adoptive cellular therapy (tumor-infiltrating lymphocyte therapy), to reduce severe toxic effects and optimize the therapeutic impact [3,18]. To this end, research is also focusing on studying genetically modified or mutant IL-2 that can target specific T cell subsets with fewer side effects [15].

As previously mentioned, numerous studies have demonstrated that IL-2 therapy acts on anti-tumor immune cells. However, a direct influence of IL-2 on RCC cells has yet to be defined. Therefore, this project’s main goal involved investigating the potential interaction of IL-2 on renal cancer cells; specifically, we aimed to determine the role of IL-2 in cell death, proliferation and survival of four human RCC cell lines: A-498, ACHN, Caki-1, and Caki-2. We selected these cell lines because they derive from the most common RCC subtypes. A-498 and Caki-1 belong to the ccRCC subtype, whereas ACHN is classified under the pRCC subtype. Initially, Caki-2 was defined as a primary ccRCC cell line; several publications have subsequently suggested that it belongs rather to the primary pRCC subtype [19].

## 2. Materials and Methods

Our project focused initially on the expression analysis of the IL-2Rα, IL-2Rβ, and IL-2Rγ subunits. For this purpose, we employed standard techniques to study the expression at various levels. Polymerase chain reaction and agarose gel electrophoresis were used to investigate the expression of the three IL-2R subunits at the transcriptional level. Western blotting and flow cytometry assays were instead performed to analyze the expression at the translational level. In particular, flow cytometry analysis was necessary to identify the expression site [20,21,22].

Subsequently, additional standard techniques were applied to investigate the functionality of the IL-2R by culturing the renal cancer cells in the presence or absence of rhIL-2. The effect of rhIL-2 on cell proliferation and survival was investigated using the BrdU incorporation assay and the Cell Proliferation Assay (MTT) [23,24]. Cell death was assessed using the Live-or-Dye fixable viability stain, which selectively penetrates cells with damaged membranes [25]. Flow cytometry assays were also used to quantify cell death via Annexin V-FITC and Propidium Iodide (PI) staining [26,27]. The cell cytotoxicity was analyzed by measuring the level of lactate dehydrogenase (LDH) [28]. Finally, to identify the apoptotic markers, cleaved PARP, and cleaved caspases 3 and 9, we employed Western blotting [29].

### 2.1. Cell Lines

The human RCC cell lines A-498, ACHN, Caki-1, and Caki-2 (purchased from ATCC, see Table 1 for details) were cultured in Dulbecco’s Modified Eagle Medium (DMEM, high glucose; Gibco, ThermoFisher Scientific, Waltham, MA, USA) supplemented with 10% Fetal Bovine Serum (P221002, Pan Biotech, Aidenbach, Germany) and 1% antibiotic (penicillin/streptomycin solution, CP23-6318, Capricorn Scientific GmbH, Ebsdorfergrund, Germany). RCC cells were cultured in 2D (monolayer) conditions.

Peripheral Blood Mononuclear Cells (PBMCs) were isolated, according to the standard Ficoll density gradient protocol (Ficoll-paque, Sigma-Aldrich, Merck, Darmstadt, Germany), from buffy coats of anonymous healthy donors obtained from the Swiss Red Cross Blood Bank, (Interregionale Blutspende, Bern, Switzerland) and Jurkat cells (purchased from ATCC; kindly provided by Prof. Michael Walch, University of Fribourg) were used as control cells for certain experiments, as explained in the corresponding sections.

### 2.2. Polymerase Chain Reaction and Agarose Gel Electrophoresis Assay

Cellular RNA was isolated from A-498, ACHN, Caki-1, and Caki-2 cells using TRI Reagent^®^ (Molecular Research Center, Inc., Cincinnati, OH, USA). Total RNA (1 µg) was reverse transcribed with GoScript^TM^ Reverse Transcription Mix, Random Primers (Promega, Madison, WI, USA), according to the manufacturer’s instructions. Polymerase chain reactions (PCRs) were performed on BioRad CFX96 Real-Time Thermal Cyclers using SensiFAST SYBR^®^ No-ROX Kit (Meridian Bioscience^TM^, Cincinnati, OH, USA) in a 20 µL reaction system. The following PCR primers were used: IL-2Rα- 5′-AAT GCA AGA GAG GTT TCC GC-3′ (F), 5′-ACT GGC TGC ATT GGA CTT TG-3′ (R); IL-2Rß- 5′-CCC ATC TCC CTC CAA GTT GT-3′ (F), 5′-ACC CGC ACC TGA AAC TCA TA-3′ (R); IL-2Rγ- 5′-CCT AGT GTG GAT GGG CAG AA-3′ (F), 5′-GGC TTC CAA TGC AAA CAG GA-3′ (R); IL-2- 5′-AGA ATC CCA AAC TCA CCA GGA-3′ (F), 5′-TGC TGA TTA AGT CCC TGG GT-3′ (R); GAPDH- 5′-AGG TCG GAG TCA ACG GAT TT-3′(F), 5′-TGA CAA GCT TCC CGT TCT CA-3′ (R); ß-Actin- 5′-CAT CCG CAA AGA CCT GTA CG-3′ (F), and 5′-CCT GCT TGC TGA TCC ACA TC-3′ (R). The values for each gene were normalized to GAPDH and ß-Actin. The results were represented as relative mRNA expression data calculated according to the ∆∆Ct method.

Human IL-2Rα, IL-2Rß, IL-2Rγ, IL-2, GAPDH, and ß-Actin cDNA product amplified by PCR were also run on agarose gels obtained by the mix of AgaPure^TM^, Agarose LE (Canvax, Boecillo, Valladolid, Spain) supplemented with Buffer TAE then with GelRed^®^ Nucleic Acid Stain (Axonlab, Aargau, Switzerland) (1:20). For loading DNA samples on the gel, we used DNA Loading Buffer Blue (Biotium, Fremont, CA, USA) (6×). Gel documentation was performed with the G: Box Imaging System (Syngene, Cambridge, UK).

### 2.3. Western Blotting

Cell pellets of A-498, ACHN, Caki-1, and Caki-2 cells were lysed in radioimmunoprecipitation assay (RIPA) buffer supplemented with protease cocktail tablets (cOmplete tablets, Roche Diagnostic GmbH, Basel, Switzerland). The protein concentration was determined using the Pierce^TM^ BCA Protein Assay Kit (ThermoFisher Scientific, Waltham, MA, USA). Proteins (50 µg) were separated on an SDS-PAGE (12%) gel and transferred onto activated polyvinylidene fluoride membranes (PVDF) using the Trans-Blot Turbo Transfer System (Bio-Rad). Following the transfer, the membranes were blocked in 5% bovine serum albumin (BSA) dissolved in TBST buffer (50 mM Tris pH 7.5, 150 mM NaCl, and 0.05% Tween-20) and incubated at 4 °C overnight under rotation with primary antibodies (Table 2). After washing with TBST, the membranes were incubated with horseradish peroxidase (HRP)-conjugated secondary antibody (Table 2) for 2 h at RT. After washing again, the SuperSignal™ West Atto Ultimate Sensitivity Substrate (ThermoFisher Scientific, Waltham, MA, USA) was used to visualize the protein bands. Images were acquired using the G: Box Imaging System (Syngene, Cambridge, UK).

Western blotting was also performed to analyze the expression of apoptotic markers, such as cleaved PARP (Asp214), cleaved caspase 3, and cleaved caspase 9. Renal cancer cell lines were cultured under three different conditions for 4 h: untreated cells (negative control), cells treated with recombinant human IL-2 IS of research grade (1 μg/mL), and cells treated with Staurosporine (1 μg/mL, positive control). Protein extraction, quantification, separation, and transfer to membranes were performed as previously described for the analysis of the expression of the three IL-2R subunits. The PVDF membranes were incubated at 4 °C overnight under rotation with primary antibodies (Table 3). After washing with TBST, the membranes were incubated with anti-rabbit IgG and HRP-linked antibody (Table 3) for 2 h at RT. After washing again, the SuperSignal™ West Atto Ultimate Sensitivity Substrate (ThermoFisher Scientific, Waltham, MA, USA) was used to visualize the protein bands. Images were acquired using the G: Box Imaging System (Syngene, Cambridge, UK).

### 2.4. Flow Cytometry Assay

RCC cells were incubated overnight with or without recombinant human IL-2 IS of research grade (1.16 μg/mL) (Miltenyi Biotec, Bergisch Gladbach, Germany). The cells were washed with FACS buffer (PBS, FBS 3%, EDTA 1 mM), after which they were incubated with a panel of fluorescence-labeled mAbs (Table 4) and Western blot assay antibodies (Table 5) for 30 min on ice in the dark. Cells binding Western blot assay antibodies were then incubated with the second antibody for 20 min on ice in the dark. The cells were washed and resuspended in FACS buffer. Data were then acquired via the BD FACSCantoII cytometer (Becton Dickinson, Franklin Lakes, NJ, USA), and data analysis was performed using FlowJo^TM^ Software V10.9 (BD Biosciences, San Jose, CA, USA).

Flow cytometry analysis was also conducted to assess the IL-2R functionality in the four human RCC cell lines.

The cancer cells were incubated for 4 h with or without recombinant human IL-2 IS of research grade (2 μg/mL) (Miltenyi Biotec, Bergisch Gladbach, Germany). Subsequently, the cells were washed with the binding buffer and incubated with Annexin V-FITC (5 μL/tube, 1:20) (Miltenyi Biotec, Bergisch Gladbach, Germany) for 15 min in the dark at RT. Cells were then washed with the binding buffer. Immediately before the acquisition of the data via the BD FACSCantoII cytometer (Becton Dickinson, Franklin Lakes, NJ, USA), PI solution (2.5 μL/tube, 1:200) (Miltenyi Biotec, Bergisch Gladbach, Germany) was added to the cells. Data analysis was performed using FlowJo^TM^ Software V10.9 (BD Biosciences).

### 2.5. Cell Proliferation Assay

Cell Proliferation ELISA, BrdU (colorimetric) (Roche Diagnostic GmbH, Basel, Switzerland) was used to determine cell proliferation of A-498, ACHN, Caki-1, and Caki-2 cell lines cultured with recombinant human IL-2 IS of research grade (Miltenyi Biotec, Bergisch Gladbach, Germany). Cells (50,000/well) were cultured in quadruplicate in 24-well plates and supplemented with BrdU Labeling Reagent (10 μL/well, 1:1000) and rhIL-2 (1 μg/mL). After 24 h of incubation, we continued the proliferation assay according to the manufacturer’s instructions. The amount of synthesized DNA incorporating the thymidine analog BrdU was detected using the microplate reader Multiskan SkyHigh (ThermoFisher Scientific, Waltham, MA, USA) at 370 nm.

### 2.6. Cell Viability Assay

CellTiter 96^®^ Non-Radioactive Cell Proliferation Assay (MTT) (Promega, Madison, WI, USA) was used to determine cell viability of A-498, ACHN, Caki-1, and Caki-2 cell lines cultured with human IL-2 IS of research grade (Miltenyi Biotec, Bergisch Gladbach, Germany). Cells (160,000/well) were cultured in quadruplicate in 24-well plates, treated with rhIL-2 (2 μg/mL, 0.4 μg/mL, and 0.08 μg/mL), and incubated for 2 h. After treatment, cells were incubated with dye solution (37.5 μL/well) for 4 h. Subsequently, solubilization/stop solution (250 μL/well) was added. The absorbance was measured at 570 nm using Multiskan SkyHigh (ThermoFisher Scientific, Waltham, MA, USA) after 5 min, 1 h, and overnight.

Live-or-Dye^TM^ Fixable Viability Staining Kit (Biotium, Fremont, CA, USA) was also used to investigate the viability of the four RCC cell lines cultured with human IL-2 IS of research grade (Miltenyi Biotec, Bergisch Gladbach, Germany). Cells (80% confluency) were cultured with rhIL-2 (0.2 μg/mL and 2 μg/mL) for 4 h, after which they were supplemented with Fixable Dead Cell Dye 32004A (488/515, 1:1000) and incubated for 30 min at RT protected from light. Cells were then fixed with 3% sucrose 2% mix and incubated overnight at 4 °C. The pictures obtained with the confocal microscope were analyzed with Fiji v10 (software).

### 2.7. Cell Cytotoxicity Assay

The cytotoxic effect of human IL-2 IS of research grade Miltenyi Biotec, Bergisch Gladbach, Germany) on A-498, ACHN, Caki-1, and Caki-2 cell lines was evaluated by measuring the level of lactate dehydrogenase (LDH) using the Cytotoxicity Detection Kit^PLUS^ following the manufacturer’s protocol (Roche Diagnostic GmbH, Basel, Switzerland). Cells (50,000/well) were cultured in quadruplicate in 24-well plates and incubated overnight with rhIL-2 (1 μg/mL and 2 μg/mL). The cells (positive control) were lysed using 50 μL of the lysis solution provided in the assay kit for 15 min at RT. A total of 100 μL of cells was collected from each sample. The supernatant was incubated with a freshly prepared reaction mixture (100 μL/well) at RT for 30 min. Subsequently, stop solution (50 μL/well) was added, and the microplate was shaken for 10 sec. Absorbance was measured at 490 nm using Multiskan SkyHigh (ThermoFisher Scientific, Waltham, MA, USA).

### 2.8. Statistical Analysis

Prism 10 software (GraphPad Software, San Diego, CA, USA) was used for statistical analysis. The threshold for statistical significance was set at *p* < 0.05. Results are reported as means ± SEM and were subjected to comparison analysis between group means by the *t*-test or Mann–Whitney test. The Shapiro–Wilk test was used to assess whether the datasets were normally distributed (*p* ≥ 0.05).

## 3. Results

### 3.1. IL-2Rα, IL-2Rβ, and IL-2Rγ Expression in A-498, ACHN, Caki-1, and Caki-2 Cell Lines

**Figure 1:**
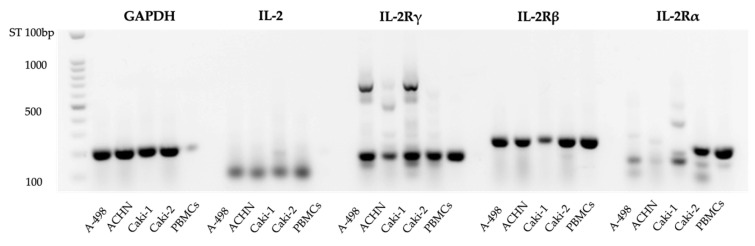
Expression of IL-2Rα, IL-2Rβ, and IL-2Rγ subunits in renal cell carcinoma cells, demonstrated by PCR products on an agarose gel. Representative blot with relative quantification shows IL-2Rα, IL-2Rβ, and IL-2Rγ protein detection in A-498, ACHN, Caki-1, and Caki-2 cancer cell lines (Figure S4). PBMCs: Peripheral Blood Mononuclear Cells, which are the control cells.

First, we investigated the rhIL-2 receptor subunit expression on RCC by visualizing the PCR products of the corresponding mRNA on agarose gels. This approach, as shown in Figure 1, has revealed the expression of the IL-2Rα gene in Caki-2 and of the IL-2β and IL-2Rγ genes in A-498, ACHN, Caki-1, and Caki-2, in agreement with the PBMC control. The expression of the IL-2Rα was also detected in the first three RCC cell lines but its molecular weight did not correspond to the control, due to a possible splice variant.

**Figure 2:**
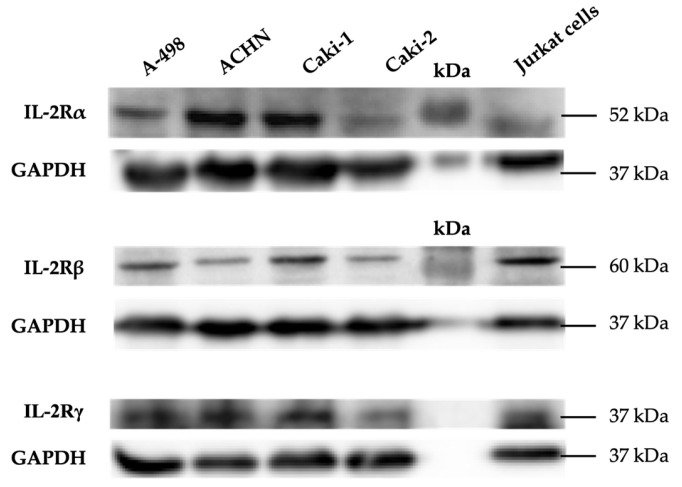
Protein expression of IL-2Rα, IL-2Rβ, and IL-2Rγ subunits in renal cell carcinoma cells, visualized by Western blot analysis. Representative blot shows IL-2Rα (Figure S1), IL-2Rβ (Figure S2), and IL-2Rγ (Figure S3) protein detection in A-498, ACHN, Caki-1, and Caki-2 cancer cell lines. Jurkat cells are the control cells.

We subsequently performed Western blot experiments to confirm that not only the mRNAs but also the proteins of the IL-2R subunits were expressed in the four RCC cell lines (Figure 2). Our results showed the glycosylated form of the IL-2Rα subunit with an apparent molecular weight of approximately 52 kDa, the unglycosylated form of the IL-2Rβ subunit at around 60 kDa, and the unglycosylated form of the IL-2Rγ subunit at approximately 37 kDa.

Once the expression of IL-2Rα, IL-2Rβ, and IL-2Rγ subunits was demonstrated on A-498, ACHN, Caki-1, and Caki-2 cell lines at both the transcriptional and translational levels, our interest was focused on examining the site of expression of these protein receptors. Indeed, the Western blot results and the PCR products indicated the expression of the IL-2Rα, IL-2Rβ, and IL-2Rγ subunits, but they did not clarify whether these subunits were expressed within the cell or on the plasma membrane. For this purpose, we decided to perform flow cytometry assays. The positive results obtained through this technique would have demonstrated the expression of the IL-2R at the plasma membrane level.

**Figure 3:**
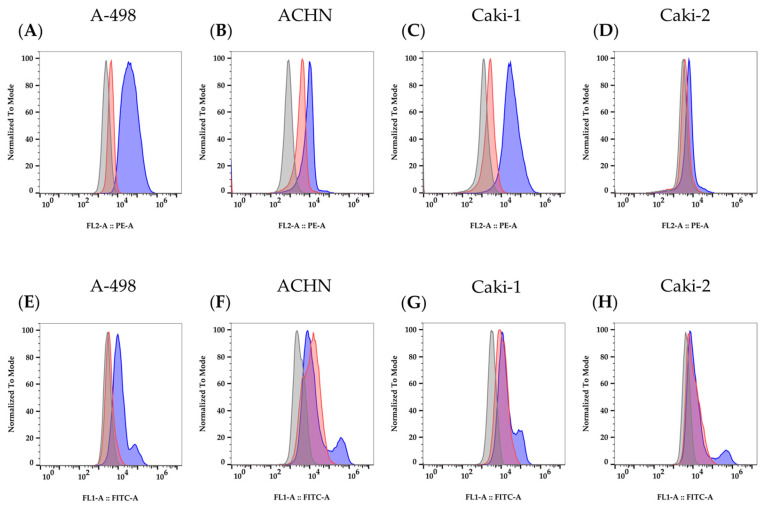
Representative flow cytometry histograms of the IL-2Rγ and IL-2Rβ surface expression for the four RCC cell lines tested. Graphs (**A**–**D**) show IL-2Rγ expression, and graphs (**E**–**H**) show IL-2Rβ expression (dark blue curves) with isotype controls (red curves) and unstaining controls (gray curves) in A-498, ACHN, Caki-1, and Caki-2 cell lines.

We obtained positive results by using specific fluorochrome-labeled antibodies that showed the expression of IL-2Rγ on the four cell lines (Figure 3A–D). The anti-IL-2Rβ (C-10) antibody, used in Western blot assays, allowed the detection of the IL-2Rβ subunit expression on the surface membrane of the renal cancer cells (Figure 3E–H). Conversely, the expression of the IL-2Rα subunit could not be detected by flow cytometry.

These results suggested that rhIL-2, once bound to its receptor, could trigger signaling pathways, thus affecting the gene expression in the RCC cells. To effectively confirm this, our study then focused on examining the functionality of IL-2Rα, IL-2Rβ, and IL-2Rγ subunits.

### 3.2. IL-2 Receptor-Mediated Response Following IL-2 Exposure of A-498, ACHN, Caki-1, and Caki-2 Cell Lines

The investigation of IL-2R functionality on the RCC cell lines was first performed using the BrdU incorporation assay, an accurate method for detecting cell proliferation via direct measurement of new DNA synthesis and corresponding cell cycling.

**Figure 4:**
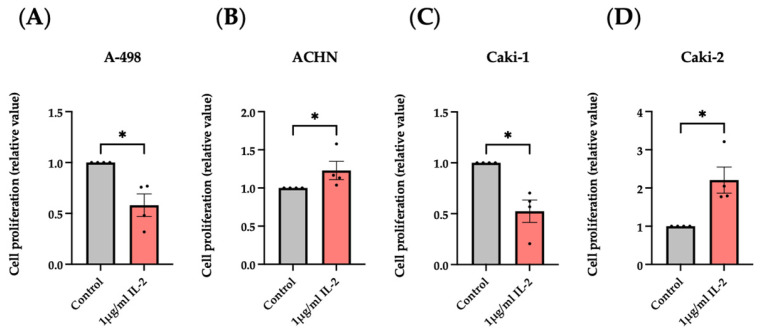
Influence of rhIL-2 on RCC cell lines analyzed by BrdU incorporation assay. Histogram bars show the variations in the cell proliferation of A-498 (**A**), ACHN (**B**), Caki-1 (**C**), and Caki-2 (**D**) cell lines cultured overnight with rhIL-2 (1 μg/mL). * *p* < 0.05 versus control. *n* = 4.

As shown in Figure 4, 1 μg/mL of rhIL-2 induced a significant decrease in the BrdU incorporation in the clear cell RCC cell lines, A-498, and Caki-1. Conversely, when cultured with the same concentration of rhIL-2, the ACHN and Caki-2 cell lines, which belong to the papillary RCC subtype, manifested an increase in cell proliferation, suggesting that the impact of rhIL-2 may depend on the RCC subtypes.

**Figure 5:**
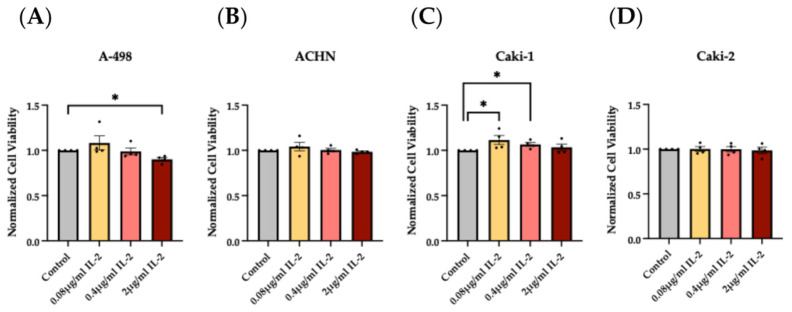
Effects of rhIL-2 on RCC cell lines analyzed by MTT viability assay. Histogram bars show the variations in the cell viability of A-498 (**A**), ACHN (**B**), Caki-1 (**C**), and Caki-2 (**D**) cell lines cultured with increasing concentrations of rhIL-2 (0.08 μg/mL, 0.4 μg/mL, and 2 μg/mL) over a 2-h period. * *p* < 0.05 versus control. *n* = 8.

MTT viability tests of the four RCC cell lines with 2 μg/mL rhIL-2 revealed that viability significantly decreased in the A-498 cells. Conversely, 0.08 μg/mL and 0.4 μg/mL of rhIL-2 positively enhanced the cell viability of the Caki-1 cell line, indicating a possible proliferative effect on these cells. A total of 0.08 μg/mL of rhIL-2 displayed a similar trend on A-498 cells. The remaining results did not show significant differences versus control. Despite rhIL-2 manifesting an opposite reaction on the cell viability and proliferation of A-498 and Caki-1 cell lines and no significant alterations on the ACHN and Caki-2 cell lines, we concluded an effect of IL-2 on the RCC cells, depending on the cell line and on the IL-2 concentration (Figure 5).

**Figure 6:**
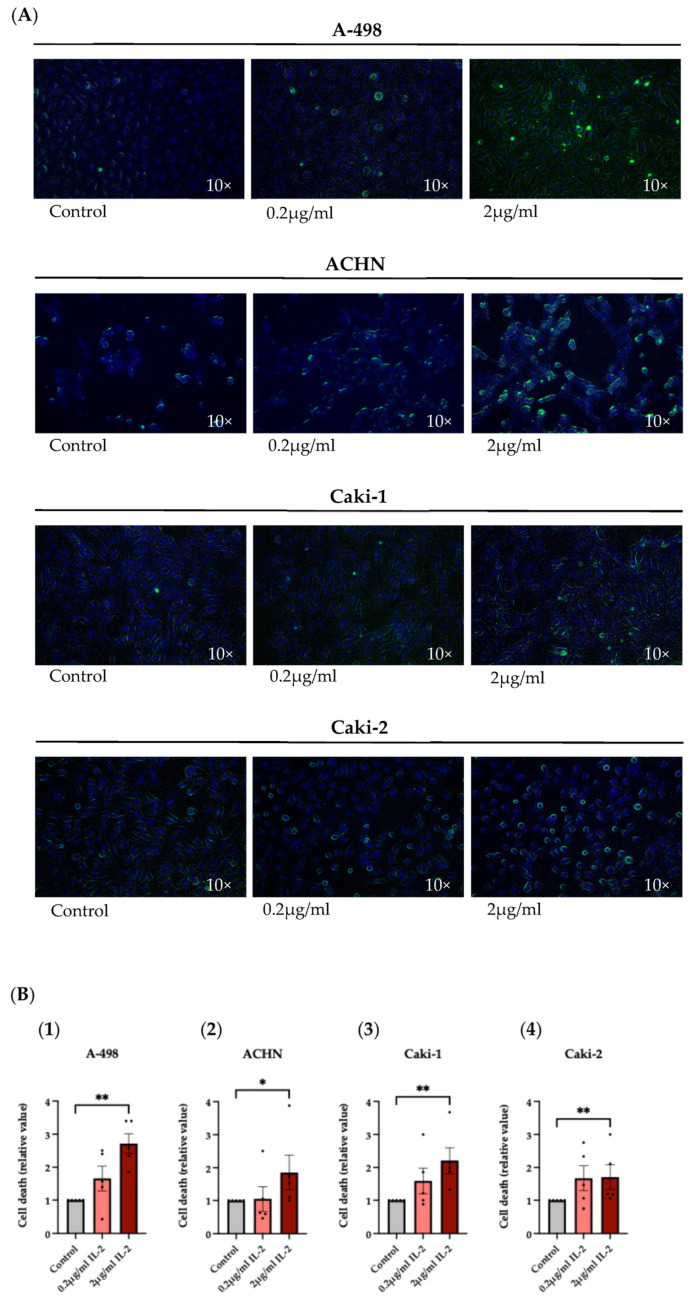
Discrimination of live and dead cells in fluorescence microscopy using Live-or-Dye^TM^ Fixable Viability Stain (Live-or-Dye 488/515). (**A**) Dead RCC cells show very bright Live-or-Dye fluorescence staining compared to live cells, allowing the two populations to be clearly distinguished. (**B**) Histogram bars show the variations in the cell death of A-498 (**1**), ACHN (**2**), Caki-1 (**3**), and Caki-2 (**4**) cell lines cultured with rhIL-2 (0.2 μg/mL and 2 μg/mL) over 4 h. * *p* < 0.05 versus control. ** *p* < 0.01 versus control. *n* = 5.

The subsequent assay involved the evaluation of cell death through the application of a Live-or-Dye^TM^ Fixable Viability Stain, a fluorescent dye able to label dead cells, on A-498, ACHN, Caki-1, and Caki-2 cell lines cultured with 0.2 μg/mL and 2 μg/mL of rhIL-2, which revealed an increase in cell death in the four RCC cell lines. As shown in the confocal microscope images (Figure 6A) and the statistical analysis (Figure 6B), this aspect was particularly evident when the RCC cells were exposed to 2 μg/mL of rhIL-2.

**Figure 7:**
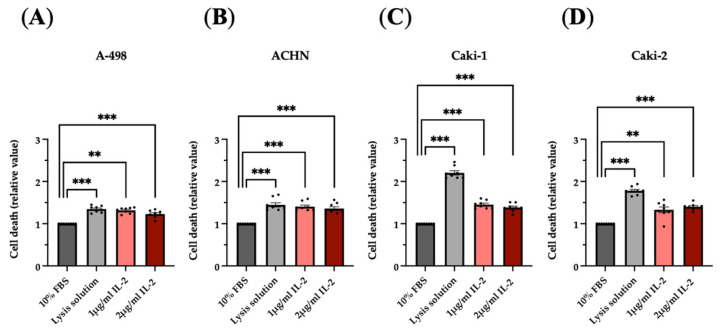
rhIL-2-induced cell death of the renal cell carcinoma lines assessed by the LDH assay. Histogram bars show the variation in the cell death of A-498 (**A**), ACHN (**B**), Caki-1 (**C**), and Caki-2 (**D**) cell lines cultured overnight with rhIL-2 (1 μg/mL and 2 μg/mL). The 10% FBS and the lysis solution were used as negative and positive controls, respectively. ** *p* < 0.01 versus control. *** *p* < 0.001 versus control. *n* = 8.

LDH assay results further confirmed the efficacy of IL-2 in eliminating renal tumor cells. Lactate dehydrogenase released from the cell’s cytoplasm, as a result of cell membrane damage and lysis of the cells, was indeed higher in the supernatant of the RCC cells cultured with 0.2 μg/mL and 2 μg/mL compared to the negative control (Figure 7).

**Figure 8:**
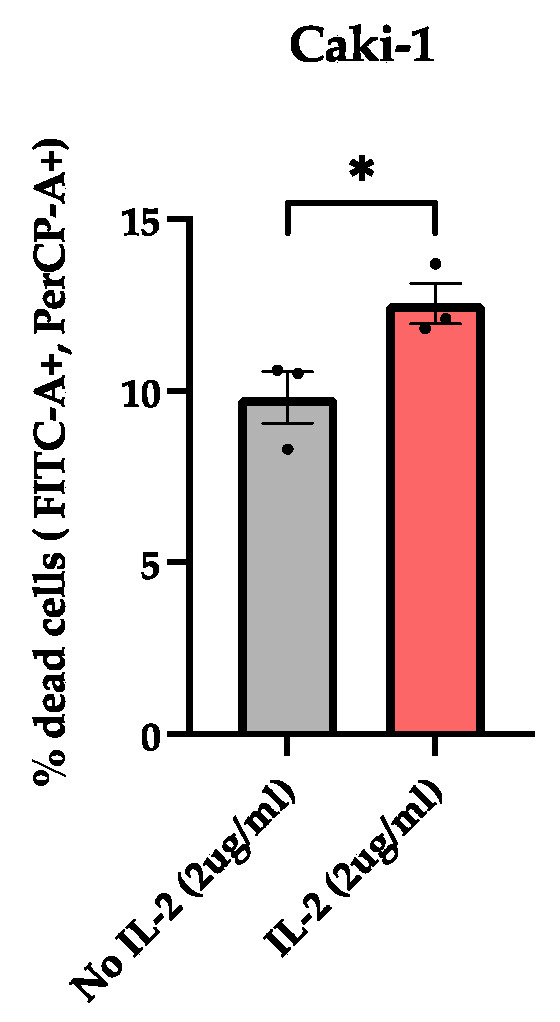
rhIL-2-induced cell death of the Caki-1 renal cell carcinoma line assessed by PI solution. Histogram bars show the variation in the cell death of the Caki-1 cell line cultured with rhIL-2 (2 μg/mL) over 4 h. * *p* < 0.05 versus control. *n* = 3.

Flow cytometry analysis using Propidium Iodide (PI) staining, a fluorescent dye that penetrates through the damaged membrane and labels the DNA of cells, further confirmed the impact of IL-2 on cell death. Notably, the Caki-1 cell line cultured with 2 μg/mL of rhIL-2 exhibited a significant increase in cell death compared to untreated controls (Figure 8).

**Figure 9:**
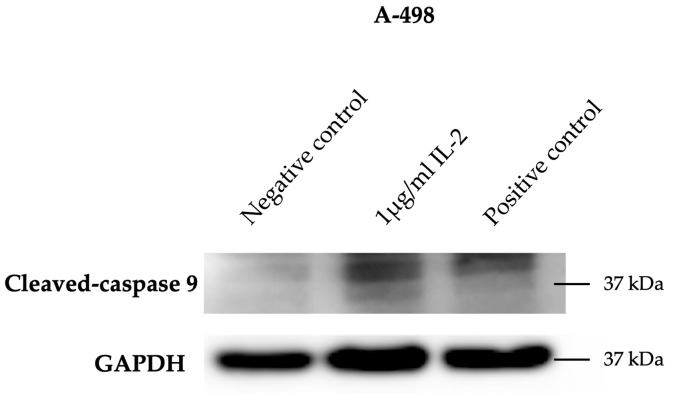
Cleaved caspase 9 expression in the A-498 renal cell carcinoma, visualized by Western blot analysis. Representative blot shows increased expression of cleaved caspase 9 protein in the A-498 cell line cultured with rhIL-2 (1 µg/mL) for 4 h, compared to negative control (untreated cells). A-498 cells cultured with Staurosporine (1 µg/mL) acted as the positive control (Figure S5).

Subsequent Western blot experiments, conducted to investigate the potential effect of IL-2 on the apoptotic pathway, revealed an increased expression of cleaved caspase-9, with a molecular weight of 37 kDa, in the A-498 cell line cultured with 1 µg/mL of rhIL-2 compared to the negative control (Figure 9).

## 4. Discussion

Numerous studies have demonstrated the involvement of HD IL-2 therapy in the stimulation of anti-tumor immune cells, which are able to counteract tumor progression. However, a direct influence of IL-2 on cancer cells has not been reported yet. In this study, we therefore aimed to investigate the expression of the IL-2 receptor in RCC cells and test for direct effects on RCC cells. For that purpose, four human RCC cell lines were used, belonging to clear cell RCC and/or papillary RCC subtypes.

We were able to confirm gene expression of the IL-2Rα, IL-2Rβ, and IL-2Rγ subunits at mRNA and protein levels in A-498, ACHN, Caki-1, and Caki-2 cell lines.

The additional protein expression analysis of the IL-2Rα, IL-2Rβ, and IL-2Rγ subunits, performed with flow cytometry, demonstrated that IL-2Rβ and γ were expressed on the cell surface of all investigated RCC cell lines. The absence of outcomes with CD25-APC (fluorescence-labeled mAb) and human CD25/IL-2Rα (Western blot assay Ab) may suggest that IL-2Rα is intracellularly expressed but not exposed on the cell surface under standard culture conditions.

The subsequent analysis of IL-2R functionality, performed using the BrdU incorporation assay, MTT viability assay, LDH release assay, Live-or-Dye™ Fixable Viability Stain, flow cytometry to quantify PI incorporation, and Western blotting to detect cleaved caspase-9, allowed us to distinguish the cellular effects mediated by IL-2 through its receptor interaction. Specifically, increased BrdU incorporation indicated that IL-2 (1 μg/mL) may enhance cell cycling and, thus, proliferation in ACHN and Caki-2 cells, whereas decreased BrdU incorporation indicated that IL-2 (1 μg/mL) may decrease cell cycling or even induce cell death in A-498 and Caki-1 cells. These results also showed how the influence of IL-2 may depend on the RCC subtypes. An additional BrdU assay has reported that this influence may be IL-2 concentration dependent, as well. Since the different pathways activated by the binding of IL-2 to its receptor expressed on the surface of the cancer cells are not yet known, to clarify these observations, a new project should be started.

A decrease in viability of A-498 cells, observed through MTT assays, indicated that IL-2 (2 μg/mL) compromises mitochondrial function. Opposite results were instead obtained by the culture of Caki-1 cells with IL-2 (0.08 μg/mL and 0.4 μg/mL), indicating that IL-2 may also positively influence the viability and proliferation of the cancer cells. In this case, it would be interesting to analyze the pathways activated by the binding of IL-2 with its receptor on the Caki-1 surface membrane to clarify the IL-2 protective effect on this cell line. Although A-498 and Caki-1 cells cultured with IL-2 indicated opposite effects on viability, they demonstrated that the clear cell RCC subtype is sensitive to IL-2. Conversely, the papillary RCC category appeared to be more resistant to the action of IL-2 and, therefore, was probably capable of repairing the lesion induced by it. These observations indicated again that the IL-2 influence may vary from the RCC subtypes, suggesting that IL-2 can possess distinct pathways for each subtype.

Cell death assays, including LDH release measurement, Live-or-Dye™ Fixable Viability Staining, and flow cytometric analysis of PI incorporation, provided insights into the effects of IL-2 on cell membrane integrity. A significant increase in the Live-or-Dye^TM^ Fixable Viability Stain incorporation, indicating an increase in surface membrane damage, was evident in the four cell lines cultured with IL-2 (2 μg/mL). IL-2-dependent cell death was also confirmed by the increased release of the stable cytoplasmic enzyme LDH into the supernatant of A-498, ACHN, Caki-1, and Caki-2 cells cultured with IL-2 (1 μg/mL and 2 μg/mL). Additionally, flow cytometry analysis revealed a significant increase in PI incorporation in the Caki-1 cell line cultured with 2 µg/mL of rhIL-2, further supporting the influence of IL-2 on the induction of cell death. The final analysis of cleaved caspase-9 expression by Western blot showed an increased level of this apoptotic marker in the A-498 cell line cultured with 1 µg/mL of rhIL-2. Combined with the MTT assay results, this finding supports the classification of cell death in the A-498 cell line as apoptotic. Several studies support this concept in other cell lines (e.g., HepG2). Indeed, it has been shown that IL-2 treatment can induce mitochondrial stress, leading to the activation of caspase-9 and, consequently, apoptosis [30].

Overall, the results reveal a clear difference in cell survival and death across the various concentrations of rhIL-2. Further research is required to investigate the signaling pathways triggered by IL-2-receptor binding in tumor cells in order to better understand the basis of these effects.

Compared to cancer cells, much more is known about IL-2Rα, IL-2Rβ, and IL-2Rγ subunit expression and function in immune cells (e.g., Jurkat cells as an experimental cell line representing T lymphocytes). Several studies have demonstrated that the high-affinity between IL-2 and its receptor is attributable to the IL-2Rα subunit; however, IL-2Rα is not required for signal transduction. Instead, signaling is ensured by the IL-2Rβ and IL-2Rγ subunits [31]. Notably, IL-2Rβ is shared by both IL-2 and IL-15, while the IL-2Rγ subunit represents a common element identified in many cytokine receptor complexes, including those for IL-2, IL-4, IL-7, IL-9, IL-15, and IL-21 [32,33].

IL-2R complex can activate the JAK-STAT, PI3K/Akt/mTOR, and the MAPK/ERK pathways, especially in immune cells. The JAK-STAT pathway is crucial for the functional maturation of T and B lymphocytes and for regulating the expression of several target genes, including IL-2 itself. The PI3K/Akt/mTOR pathway promotes lymphocyte survival, proliferation, and metabolism, whereas the MAPK/ERK pathway regulates cell cycle progression and differentiation processes [34,35]. However, little is known about activation of those pathways in cancer cells, especially in RCC.

## 5. Conclusions

In conclusion, we can affirm that the expression and functionality of the IL-2Rα, IL-2Rβ, and IL-2Rγ subunits on the RCC cell lines A-498, ACHN, Caki-1, and Caki-2 have been demonstrated, thus confirming our initial hypothesis. In addition, our results showed that depending on cell line and IL-2 concentration, IL-2 may hinder cell proliferation and viability and induce damage to the cell membrane, thereby increasing cell death; however, it may also promote cell survival under certain conditions. Based on the results obtained in this study, IL-2-induced cell death cannot be clearly defined in the ACHN, Caki-1, and Caki-2 cell lines; however, in the A-498 cell line, it can be characterized as apoptotic.

Nevertheless, we still do not know about which signaling pathways may be activated or blocked upon IL-2 binding to the IL-2 receptor subunits in RCC, as well as how IL-2-concentration-dependent actions may work in cancer cells. Future research ought to elaborate on this missing knowledge. Once such information is available, IL-2 would be a reasonable therapeutic option to directly kill the susceptible renal cancer cells. Because of the heterogeneity among the different cell lines, a biopsy may be very helpful in assessing the effectiveness of IL-2 immunotherapy before its administration, as well as for the characterization of the tumor microenvironment.

## Figures and Tables

**Table 1:** Origin of the human RCC cell lines A-498, ACHN, Caki-1, and Caki-2 (according to ATCC, **19**).

Cell Line	Age	Gender	Diagnosis	Subtype
A-498	52-year-old	Female	Kidney cancer	ccRCC
ACHN	22-year-old	Male	Renal adenocarcinoma	pRCC
Caki-1	49-year-old	Male	Kidney cancer	ccRCC
Caki-2	69-year-old	Male	Kidney cancer	ccRCC/pRCC

**Table 2:** Antibodies used for Western blot analysis.

Antibody	Dilution	Company
Human CD25/IL-2Rα	1:250	R&D Systems, Minneapolis, MN, USA
IL-2Rβ (C-10): sc-393093	1:200	Santa Cruz Biotechnology, Heidelberg, Germany
IL-2Rγ (A-10): sc-271060	1:200	Santa Cruz Biotechnology, Heidelberg, Germany
GAPDH	1:5000	Proteintech, San Diego, CA, USA
HRP-conjugated goat anti-mouse immunoglobulin	1:2500	R&D Systems, Minneapolis, MN, USA

**Table 3:** Antibodies used for Western blot analysis.

Antibody	Dilution	Company
Cleaved PARP (Asp214) (D64E10) XP(R) Rabbit mAb	1:1000	Cell Signaling Technology^®^, Massachusetts, MA, USA
Cleaved Caspase-3 (D175) (5A1E) Rabbit mAb	1:1000	Cell Signaling Technology^®^, Massachusetts, MA, USA
Cleaved Caspase-9 (Asp330) (E5Z7N) Rabbit mAb	1:1000	Cell Signaling Technology^®^, Massachusetts, MA, USA
GAPDH	1:5000	Proteintech, San Diego, CA, USA
Anti-rabbit IgG, HRP-linked Antibody	1:1000	Cell Signaling Technology^®^, Massachusetts, MA, USA

**Table 4:** Fluorescence-labeled mAbs used for flow cytometry assay.

Antibody	Clone	Dilution	Company
CD25-APC	S43.10	1:50	Miltenyi Biotec, Bergisch Gladbach, Germany
CD122-FITC	REA293	1:50	Miltenyi Biotec, Bergisch Gladbach, Germany
CD132-PE	REA293	1:50	Miltenyi Biotec, Bergisch Gladbach, Germany

**Table 5:** Western blot assay antibodies used for flow cytometry assay.

Antibody	Dilution	Company
Human CD25/IL-2Rα	1:50	R&D Systems, Minneapolis, MN, USA
IL-2Rβ (C-10): sc-393093	1:50	Santa Cruz Biotechnology, Heidelberg, Germany
Alexa Fluor^®^ 488-AffiniPure F(ab’)2 Fragment Donkey Anti-Mouse IgG (H + L)	1:500	Jackson Immuno Research Laboratories, West Grove, PA, USA

## Data Availability

All data in this study were newly generated and are available from the corresponding authors for further processing.

## Supplementary Materials

The following supporting information can be downloaded at: https://www.mdpi.com/article/10.3390/cimb47100830/s1.

## Abbreviations

RCC	Renal cell carcinoma
ccRCC	Clear cell renal cell carcinoma
pRCC	Papillary renal cell carcinoma
HD IL-2 therapy	High-dose IL-2 therapy
IL-2R	Interleukin-2 receptor
IL-2Rα	Interleukin-2 receptor alpha (CD25)
IL-2Rβ	Interleukin-2 receptor beta (CD122)
IL-2Rγ	Interleukin-2 receptor gamma (CD132, γc)
rhIL-2	Recombinant human interleukin-2
IL-2	Interleukin-2
PBMCs	Peripheral Blood Mononuclear Cells
BrdU	Bromodeoxyuridine
MTT	3-(4,5-dimethylthiazol-2-yl)-2,5-diphenyltetrazolium bromide
LDH	Lactate dehydrogenase
SEM	Standard error of the mean
