# Supplementary material for: IL-2 Receptor Expression in Renal Cell Carcinoma Cells: IL-2 Influences Cell Survival and Induces Cell Death
Source: Curr Issues Mol Biol. 2025 Oct 9;47(10):830. doi: 10.3390/cimb47100830 (PMC12564725; doi:10.3390/cimb47100830)
Supplement: Supplementary file 1 [file cimb-47-00830-s001.zip › cimb-3901673-supplementary.pdf]

Supplementary Materials

Complementary images:

Western blot images:

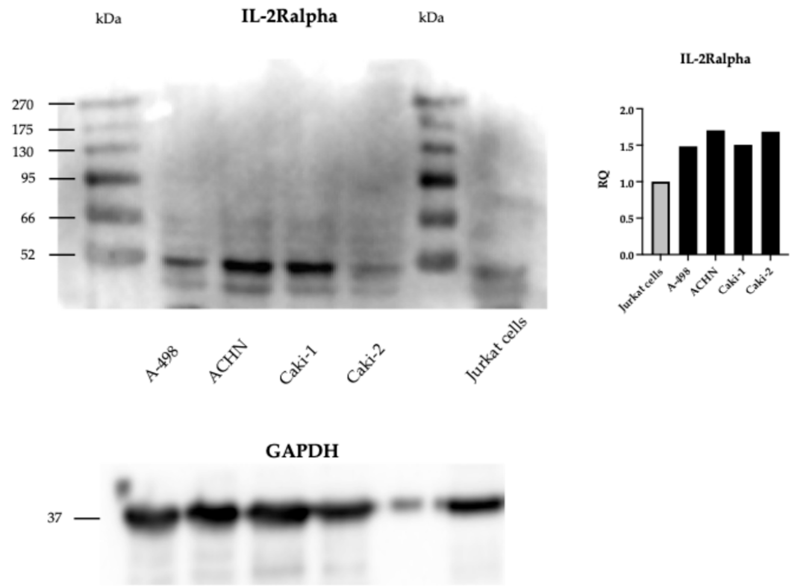

Figure S1. Protein expression of IL-2Rα subunit in renal cell carcinoma cells, analysed by Western blot. A representative blot shows IL-2Rα protein detection in A-498, ACHN, Caki-1, and Caki-2 renal cancer cell lines *with a corresponding molecular weight of approximately 52 kDa. Jurkat cells were used as a positive control. GAPDH was used as a loading control. Band intensities were quantified and are shown as relative expression levels normalised to Jurkat cells.*

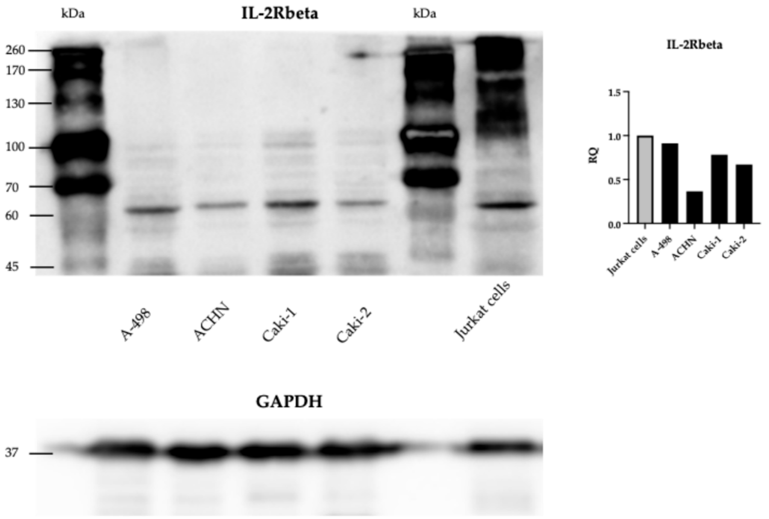

Figure S2. Protein expression of IL-2Rβ subunit in renal cell carcinoma cells, analysed by Western blot. A representative blot shows IL-2Rβ protein detection in A-498, ACHN, Caki-1, and Caki-2 renal cancer cell lines *with a corresponding molecular weight of approximately 60 kDa. Jurkat cells were used as a positive control. GAPDH was used as a loading control. Band intensities were quantified and are shown as relative expression levels normalised to Jurkat cells.*

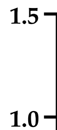

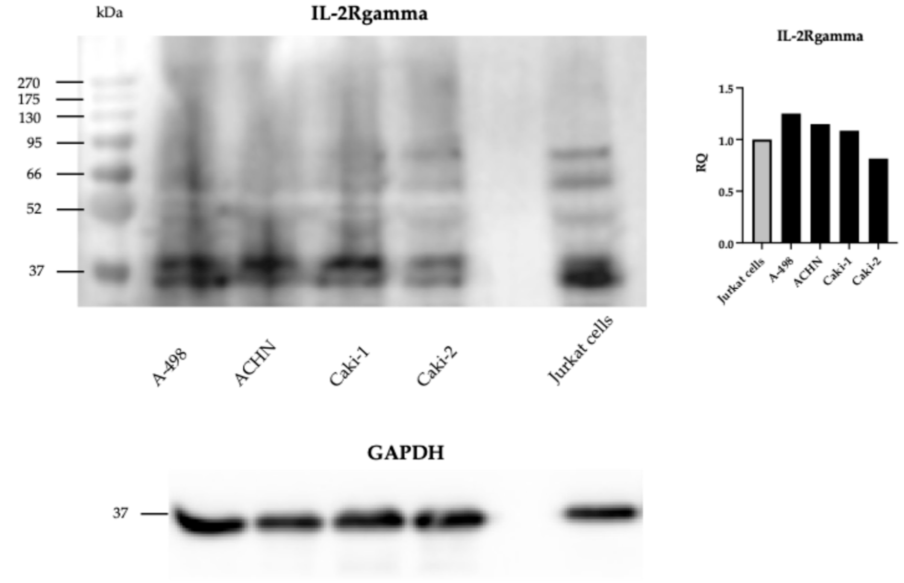

Figure S3. Protein expression of IL-2Rγ subunit in renal cell carcinoma cells, analysed by Western blot. A representative blot shows IL-2Rβ protein detection in A-498, ACHN, Caki-1, and Caki-2 renal cancer cell lines *with a corresponding molecular weight of approximately 37 kDa. Jurkat cells were used as a positive control. GAPDH was used as a loading control. Band intensities were quantified and are shown as relative expression levels normalised to Jurkat cells.*

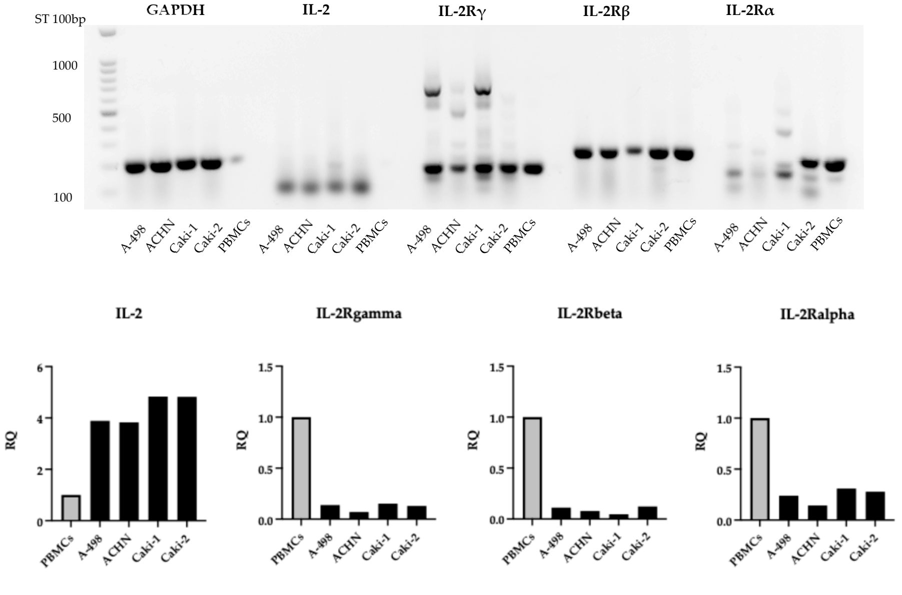

Figure S4. Expression of IL-2Rα, IL-2Rβ, and IL-2Rγ subunits in renal cell carcinoma cells, analysed by PCR products on an agarose gel. A representative gel image with relative quantification shows the expression of IL-2Rα, IL-2Rβ, and IL-2Rγ proteins in A-498, ACHN, Caki-1, and Caki-2 renal cancer cell lines. *GAPDH was used as a loading control. Band intensities were quantified and are shown as relative expression levels normalised to* PBMCs: Peripheral Blood Mononuclear Cells.

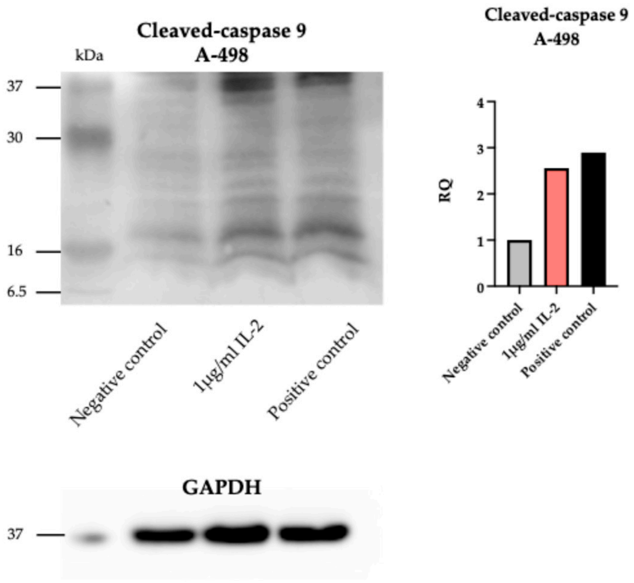

Figure S5. Cleaved-caspase 9 expression in the A-498 renal cell carcinoma, analysed by Western blot analysis. A representative blot shows increased levels of cleaved-caspase 9 (37 kDa) in the A-498 cell line cultured with rhIL-2 (1μg/ml) for 4 h, compared to the negative control (untreated cells). A-498 cells cultured with Staurosporine (1μg/ml) acted as a positive control. *GAPDH was used as a loading control. Band intensities were quantified and are shown as relative expression levels, normalised to the negative control.*
